# Bioactive Glasses: Where Are We and Where Are We Going?

**DOI:** 10.3390/jfb9010025

**Published:** 2018-03-19

**Authors:** Francesco Baino, Sepideh Hamzehlou, Saeid Kargozar

**Affiliations:** 1 Institute of Materials Physics and Engineering, Applied Science and Technology Department, Politecnico di Torino, Corso Duca degli Abruzzi 24, 10129 Torino, Italy; 2 Department of Medical Genetics, School of Medicine, Tehran University of Medical Sciences, 14155-6447 Tehran, Iran; sepidy88@hotmail.com; 3 Medical Genetics Network (MeGeNe), Universal Scientific Education and Research Network (USERN), Tehran, Iran; 4 Department of Modern Sciences and Technologies, School of Medicine, Mashhad University of Medical Sciences, P.O. Box 917794-8564, Mashhad, Iran

**Keywords:** bioglass, tissue engineering, scaffold, coating, angiogenesis, drug delivery, ion release, mesoporous bioactive glasses, in vitro, in vivo

## Abstract

Bioactive glasses caused a revolution in healthcare and paved the way for modern biomaterial-driven regenerative medicine. The first 45S5 glass composition, invented by Larry Hench fifty years ago, was able to bond to living bone and to stimulate osteogenesis through the release of biologically-active ions. 45S5-based glass products have been successfully implanted in millions of patients worldwide, mainly to repair bone and dental defects and, over the years, many other bioactive glass compositions have been proposed for innovative biomedical applications, such as soft tissue repair and drug delivery. The full potential of bioactive glasses seems still yet to be fulfilled, and many of today’s achievements were unthinkable when research began. As a result, the research involving bioactive glasses is highly stimulating and requires a cross-disciplinary collaboration among glass chemists, bioengineers, and clinicians. The present article provides a picture of the current clinical applications of bioactive glasses, and depicts six relevant challenges deserving to be tackled in the near future. We hope that this work can be useful to both early-stage researchers, who are moving with their first steps in the world of bioactive glasses, and experienced scientists, to stimulate discussion about future research and discover new applications for glass in medicine.

## 1. Introduction—The Invention of Bioactive Glass

The need to replace damaged parts of the body in order to restore their physiological functionality has always been the driving force which has supported research into the discovery and the design of new biomaterials, in order to perform this task as efficiently as possible. 

After the initial definition of biomaterial, based on the criterion of maximum biochemical/biological inertness in contact with body fluids (first-generation materials) [1], the discovery of 45S5 Bioglass^®^ by Hench in 1969 [2] constituted—for the first time in the story of biomaterials—an alternative. Since then, the concept of biocompatibility has been extended to all those materials which were able to promote a positive response of the living organism through the formation of a strong tissue-implant bond (second-generation materials) and the genetic activation of specific cell pathways (third-generation smart materials) [3].

45S5 Bioglass^®^ represents the first example of a biomaterial belonging to the third generation, thanks to the biological role of its ionic dissolution products released into the physiological environment [4]. The discovery of bioactive glasses (BGs) is attributed to Larry Hench, a Research Professor in the Department of Materials Science and Engineering at the University of Florida and then Director of the Bioglass Research Centre at the same University. As often happens when talking about scientific discoveries, the discovery of BGs also seems to be, only apparently, the result of a sequence of random events (serendipity). The foundations of this discovery are to be founded in a friendly conversation between Larry Hench and a U.S. Army colonel just returned from the Vietnam War in 1967 [5]. The topic of the talk was the rejection of polymeric and metal implants, which were used at that time for the replacement of living tissues, as they were characterized by chemical inertness. However, after being put in contact with the physiological environment, these grafting materials were surrounded by a fibrous capsule of scar tissue that compromised their integration with the host tissue. After listening to some studies about gamma rays applied to vanadia-phosphate semiconductors conducted by Hench and his coworkers, the colonel’s question was simple, but at the same time, very inspirational: “If you can make a material that will survive exposure to high energy radiation, can you make a material that will survive exposure to human body?”.

Hench was fascinated by the colonel’s question, also in the light of its social implication. In fact, once the war in Vietnam ended, the need for materials able to replace amputated limbs and compromised tissues, without them being rejected, was a matter of considerable importance, mainly aimed at the social reintegration of survivors. Hench based the so-called “hypothesis of bioactive glass” on two pillars: (i) metals and synthetic polymers elicited a “foreign body reaction” because their components were completely different from those that make up living tissues, and (ii) a material that was capable of forming a bone-like hydroxyapatite layer on its surface should not be rejected by the body, as hydroxyapatite is the main mineral phase of natural bone tissue [2]. From 1969 to 1971, Hench and his coworkers designed and studied different glass formulations based on the SiO_2_–Na_2_O–CaO–P_2_O_5_ oxide system, and they finally selected the composition 45SiO_2_–24.5Na_2_O–24.5CaO–6P_2_O_5_ (wt %), characterized by high amounts of Na_2_O and CaO, as well as a relatively high CaO/P_2_O_5_ ratio that makes the surface of the material very reactive in physiological environment [6]. This glass composition, referred to as 45S5, also had the advantage of being extremely easy to melt, due to its proximity to the ternary eutectic. The name Bioglass^®^ was then trademarked by the University of Florida as the name for the original 45S5 composition and, therefore, it should be used only with reference to that composition and not generally to indicate BGs. The studies conducted by Hench on 45S5 Bioglass^®^ have been comprehensively reviewed by Montazerian and Zanotto in a recent publication [7].

Over the last forty years, many new compositions and other types of BGs have been proposed for optimizing the body’s response according to the specific clinical applications. In addition to the silicate BGs, there are also borate glasses, which are particularly appreciated for their high dissolution rates and apatite-forming ability, and phosphate glasses, exhibiting less pronounced bioactivity but high solubility once they come in contact with biological fluids [8]. Furthermore, besides Na^+^ and Ca^2+^, it is possible to include other cations within the glass network in order to confer additional beneficial properties [9]. As an example, the addition of silver has been reported to give antimicrobial properties because the release of Ag^+^ ions during glass dissolution acts as a killing agent on several bacterial strains (e.g., *Escherichia coli*, *Pseudomonas aeruginosa*, and *Staphylococcus aureus*) without eliciting any toxic effect on human osteoblasts [10,11]. The effect of Sr-doped BGs has also been examined for the treatment of osteoporotic bone (antiresorptive action) in order to deliver a steady supply of Sr^2+^ ions to the bone defect site: in fact, the studies have shown an inhibition in osteoclastic activity as the strontium content increases [12,13]. The interested reader is addressed to a valuable work published by Jones, who summarized the evolution of research about BGs over the last decades [14]. 

## 2. Current Clinical Applications of BGs—Where Are We?

BGs are not only able to form a hydroxyapatite-like surface layer after being put in contact with biological fluids, thus promoting a stable bond to living bone (osteoconduction), but also osteoinductive, i.e., they are able to stimulate bone cells towards a path of regeneration and self-repair, thus significantly accelerating tissue healing kinetics [15]. It has been estimated that over about 30 years, from 1985 (FDA approval) to 2016, Hench’s original 45S5 Bioglass^®^ has been implanted in 1.5 millions of patients to repair bone and dental defects [16]. Other glass and glass-ceramic products have also made available to surgeons for clinical use; a summary of the main applications of BGs, along with some of today’s commercial products, is reported in Table 1. 

The first 45S5 Bioglass^®^ implant cleared for clinical use in the USA aimed to replace the small bones of the middle ear in order to treat conductive hearing losses [17]. This device received FDA approval in 1985, and was then commercialized under the name of “Bioglass^®^ Ossicular Reconstruction Prosthesis” or “Middle Ear Prosthesis” MEP^®^. This implant had a very simple design, comprising of a non-porous truncated cone of fixed size, produced by melt-quenching, which allowed sound conduction from the eardrum to the inner structures of the ear (cochlea). MEP^®^ was firmly bonded to living tissues at both its ends, because of the ability of 45S5 Bioglass^®^ to bond to calcified hard tissues (e.g., bone) and to soft collagenous tissues (e.g., eardrum) [18]. Although short-term and mid-term results revealed better performance of MEP^®^ compared to nearly-inert implants (e.g., alumina prosthetic ossicles) [19], long-term clinical studies (10 years of follow-up) showed that 45S5 Bioglass^®^ was prone to progressive dissolution and fragmentation in the biological environment of the middle ear [20]. Therefore, MEP^®^ implants were taken off the U.S. market in the early 2000s. A modified version of the original MEP^®^ implant (Douek-MED^TM^; 45S5 Bioglass^®^ cones of three different sizes) is still commercially available in some European countries. 

The middle ear small bones can be also replaced by using synthetic implants made of Ceravital^®^ glass-ceramics. In spite of the good clinical performance regarding auditory rehabilitation, the application of Ceravital^®^ in otology was debated, since the implant was prone to dissolution over time in vivo (albeit at a slower rate compared to 45S5 Bioglass^®^) [21] and, therefore, the production of such devices has currently been stopped.

45S5 Bioglass^®^ was also investigated to anchor cochlear implants to the temporal bone of profoundly deaf patients who suffered from irreversible damage to their cochlea. This device was commercialized as Bioglass^®^-EPI (extracochlear percutaneous implant) about 30 years ago. This product was a 45S5 Bioglass^®^ sleeve that bonded to the temporal bone, protruded through the skin (forming a tight bond with collagenous soft tissues, too), and acted as a percutaneous and stable seal, protecting the interior electronics [22]. Similar to MEP^®^, Bioglass^®^-EPI was taken off the market, too, in the late 1990s, as a result of the risks associated with glass dissolution over time. For this reason, the 45S5 Bioglass^®^ sleeve was replaced by a titanium peg in the next-generation design [23].

Following the promising results of MEP^®^ and Bioglass^®^-EPI, the third 45S5-based commercial device was the Endosseous Ridge Maintenance Implant (ERMI^®^), placed on the market in 1988, and still applied today in periodontal surgery. This device is a cone of 45S5 Bioglass^®^ that, after being inserted into fresh tooth extraction sites, can replace tooth roots and provide a stable support for dentures. ERMI^®^ has been used in a 5-year follow-up study involving the placement of 242 implants in 29 patients with good results, showing its high stability accompanied by a safe support to dental structures [24].

None of the products explained above is in widespread clinical use, as surgeons usually prefer more “versatile” implants that can be easily cut or shaped somehow to match the patient’s anatomy, which is impossible with rigid BG cones of fixed size. Monolithic BG is more suited to implants that are custom-made for the needs of a specific patient. An interesting example is provided by the treatment of orbital bone fractures. Thompson [25] reported the rehabilitation, from both cosmetic and functional viewpoints (5-year follow-up), of 30 patients implanted with melt-derived 45S5 Bioglass^®^ plates. In this case, the BG implants were successful where autologous bone failed. Similar results were reported in a set of consecutive studies that were carried out in Finland during a 20 year period (from the late 1980s to the early 2000s), in which melt-cast S53P4 plates (53SiO_2_–20CaO–23Na_2_O–4P_2_O_5_ wt %, BoneAlive^®^, Abmin Technologies Ltd./Vivoxid, Finland) were implanted in humans for the repair of orbital floor fractures [26,27,28]. It was reported that S53P4 BG was able to induce the growth of new orbital bone, was prone to slow dissolution without any problem regarding the mechanical integrity/support and, provided that size and shape of the BG implant were suitably selected, excellent aesthetic and functional results could be achieved. Additionally, use of a BG implant was associated to less morbidity, as neither autologous donor site nor additional harvesting surgery were needed. 

In order to meet the surgeons’ requirements, BG particles that can be easily pressed into a bone defect were made available on the market. On this matter, 45S5 Bioglass^®^ particulate was commercially developed under the trade name of PerioGlas^®^ (NovaBone Products LLC, Alachua, FL, USA) and approved by FDA in 1993. This product was sold in more than 35 countries worldwide to use for repairing jaw bone defects that occurred with periodontal disease [29]. Two specific applications of PerioGlas^®^ (particle size within 90–710 µm) are (i) the regeneration of bone around the root of a healthy tooth, with the aim of saving the tooth, and (ii) the repair of bone defects in the jaw, so that the quality and quantity of regenerated tissue becomes adequate for allowing anchorage of titanium implants [30].

NovaBone^®^ (NovaBone Products LLC) is the trade name of a 45S5 Bioglass^®^ particulate which was cleared in 1999 for repairing bone defects in maxillofacial or orthopedic non-load-bearing sites [29]. Just before being implanted, NovaBone^®^ is often mixed by the surgeon with blood from the defect or balanced salt solution to acquire a moldable consistency; thus, the resulting putty can be pressed into the defect and conform to its size and shape. In a clinical study, NovaBone^®^ was used for the treatment of idiopathic scoliosis in posterior spinal fusions surgery, and the results were compared to those obtained using iliac crest bone autograft [31]. The surgeon mixed NovaBone^®^ with the patient’s blood and secured the BG putty in place by compressing the neighboring vertebrae with metal screws and wires. The results revealed less infections (2%) and mechanical failures (2%) for NovaBone^®^ in comparison to the iliac autograft (5% and 7.5%, respectively) over a 4 year follow-up. Another 45S5-based product is Biogran^®^ (Biomet 3i, Palm Beach Gardens, FL, USA), which is mainly used for maxillofacial and dental applications (repair of defects in the jaw bone) and exhibits a narrower particle size distribution (300–360 µm) than PerioGlas^®^ [32]. 

**Figure 1:**
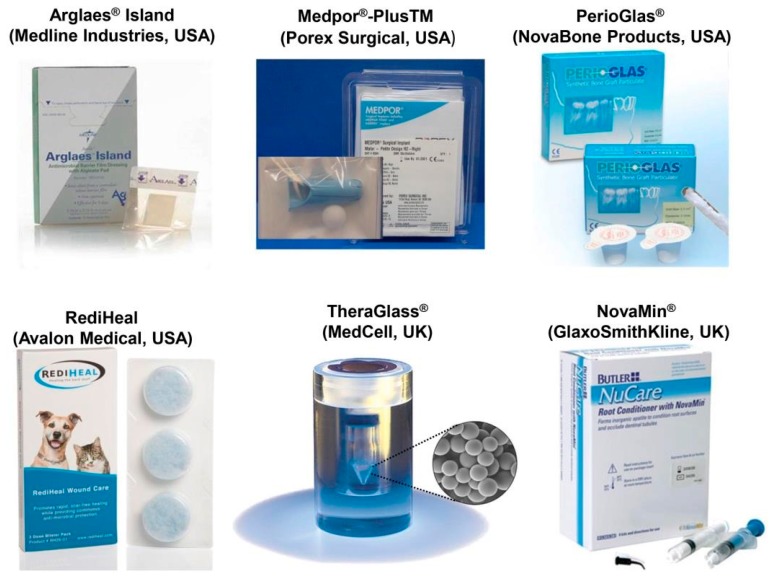
Some examples of commercially produced glasses, available on the market.

Apart from 45S5 Bioglass^®^, other FDA-approved or CE-marked BGs are available on the market in different shapes, from particulate to porous blocks (see Figure 1). Most of commercial BGs exhibit a SiO_2_-based composition, containing some additional modifiers in specific amounts for enhancing the bioactivity or imparting special characteristics to the material; for example, SrO has been introduced in StronBone^®^ (44.5SiO_2_–4Na_2_O–4K_2_O–7.5MgO–17.8CaO–4.5P_2_O_5_–17.8SrO mol %, RepRegen, London, UK) to reduce bone resorption [33,34]. The most commonly-used BGs are 13–93 (53SiO_2_–6Na_2_O–12K_2_O–5MgO–20CaO–4P_2_O_5_ wt %) and S53P4, for which a higher number of studies have been reported in the literature. Based on data from clinical trials, both these glasses in a particulate form (diameter within 0.5–1 mm) can improve the bone repair process (quality and quantity of regenerated bone) in the frontal sinus in comparison to synthetic hydroxyapatite [35]. Faster bone regeneration was observed in the case of BoneAlive^®^ compared to 13–93, probably due to the presence of MgO in the latter glass, which partially inhibits apatite-forming ability and bioactivity. BoneAlive^®^ granules (1–4 mm) were reported to be successful for the repair of large bone defects (up to 30 cm^3^) following the removal of benign bone tumor in the hand, tibia, and humerus [36]. The obliteration of surgically-created cavities in the mastoid bone is another application proposed for both 45S5 Bioglass^®^ and BonAlive^®^ particulate [37,38]. BG powder synthesized by the sol-gel process (70SiO_2_–30CaO mol %), exhibiting faster bone healing rate than melt-derived BGs, due to inherent nanoporosity, has recently been marketed for bone repair (TheraGlass^®^, MedCell, Burgess Hill, UK). 

45S5 Bioglass^®^ has also been commercialized as porous glass-ceramic sintered blocks. However, 45S5-derived glass-ceramic is less bioactive than the parent glass, as crystallization of a calcium-sodium silicate phase involves a decrease of bioactivity [39]. On the other hand, the sinterability window of 45S5 Bioglass^®^ is extremely narrow and, thus, it cannot be sintered without undergoing devitrification [40]. Other BGs, such as 13–93, have been designed to retain their amorphous state upon sintering [41]. 

45S5-based products for oral care have also been made available on the market in recent years. In 2004, a very fine 45S5 Bioglass^®^ particulate (mean size 18 µm) called NovaMin^®^ (NovaMin Technology, FL, USA; now owned by GlaxoSmithKline, Brentford, UK) was added to a toothpaste with the aim of treating dental hypersensitivity, which is currently estimated to affect about one-third of world population [42,43,44]. The function of NovaMin^®^ is the occlusion of dentinal tubules and the remineralization of the tooth surface, thus eliminating the cause of the disease [45]. This product was also used for whitening treatments of teeth. Dentists usually employ abrasive ceramic particles (e.g., sodium bicarbonate) in combination with high-pressure air flow to remove stains on the surface of tooth; however, this operation can be difficult on those patients who suffer from dental hypersensitivity. In a study, the efficacy of air polishing with NovaMin^®^ (Sylc, OSspray Ltd., London, UK) was compared to the conventional procedure (Prophy-Jet, Dentsply, York, PA, USA) Data from patients’ subjective scoring confirmed a significant reduction (44%) of tooth sensitivity for those who underwent air polishing with NovaMin^®^ [46]. Furthermore, the treatment with NovaMin^®^ seemed to impart a whiter appearance to the teeth, compared to bleaching with sodium bicarbonate.

BGs were also employed to produce glass polyalkenoate cements for use in dentistry and fixation of joint prostheses to bone. Significant research efforts were made to substitute aluminum, which was contained in the early types of glass cements, but was suspected of lethal intoxication, with other non-toxic elements. Zn-containing Al-free glass-based bone cements were developed that showed great promise exhibiting adequate mechanical properties, bone-bonding ability, and antibacterial effect [47,48,49]. In these glass networks, ZnO acts as a network modifier and not as an intermediate oxide [50]. This topic, along with other applications of Zn-doped BGs, was recently reviewed by Balasubramanian et al. [51]. 

Available literature witnesses that BGs have been mainly used for applications in contact with bone tissue; however, they have recently shown promise for the repair of soft tissues, too [52]. This emerging field of research is one of the future challenges associated with BGs, and will be also discussed in Section 3.3. BGs have attracted great interest by researchers, as their ionic dissolution products were found to stimulate angiogenesis, which plays a key role, for example, in wound healing and some ophthalmic diseases. To the best of the authors’ knowledge, at present, there exist only two BG-based commercial products having a clear angiogenic function. Biodegradable tiny cotton-candy borate BG (Mo-Sci Corp., Rolla, MO, USA), mimicking the microstructure of a fibrin clot, was reported to accelerate wound healing in both animals and human patients [53]. These BG nanofibers (basic 13-93B3 composition: 53B_2_O_3_–6Na_2_O–12K_2_O–5MgO–20CaO–4P_2_O_5_ wt %), trade-named as DermaFuse™/Mirragen™, help impressively the healing of long-term venous stasis ulcers in diabetic patients, who were irresponsive conventional treatment [54]. Studies carried out in a rat subcutaneous model revealed that the angiogenetic effect can be further improved by doping the BG with small amounts of copper that is locally released into the biological environment [55]. A commercial product, called “RediHeal” (Avalon Medical, Stillwater, MN, USA), is available in veterinarian medicine, and its FDA approval for clinical use in humans is currently pending. 

In the field of ocular surgery, 45S5-based NovaBone^®^ particles were used to coat porous polyethylene orbital implants for enucleation. Angiogenesis stimulated by BG was a key added value in this application as the growth of vascularized connective tissue inside the implant macropores (fibrovascularization) is crucial for the success of the orbital implant [56]. Investigations through magnetic resonance imagining (MRI) in human patients showed a statistically significant increase in the rate of fibrovascularization of BG-coated implants compared to the porous polyethylene alone (Medpor^®^ implant) [57,58]. This BG-coated orbital implant was cleared via the 510(k) process by FDA in 2002 and, since then, has been marketed as Medpor^®^-Plus^TM^ (Porex Surgical, Newnan, GA, USA).

There are also few other BG-based products for applications in wound healing and peripheral nerve regeneration. Resorbable Ag-doped phosphate glasses combined either with a polymeric adhesive for wound care film dressing (Antimicrobial Arglaes^®^ film and Antimicrobial Arglaes^®^ Island, Medline Industries, Northfield, IL, USA) or with alginate for topical powders (Arglaes^®^ powder, Medline Industries, USA) have been recently made available on the market, and allow a prolonged control of infections due to the sustained release of silver, which is known as a potent antibacterial agent.

A tube of resorbable Na_2_O–CaO–P_2_O_5_ glass (Corglaes^®^, Giltech Ltd., Ayr, UK) was tested for the repair of a divided facial nerve of sheep as an alternative to the end-to-end suturing [59]. The two nerve stumps were approximated inside the tube (diameter 4 mm, length 40 mm) and then sutured to it. After a 10 month follow-up, the phosphate glass tube fully dissolved, and the nerve was regenerated with a uniform diameter along its length. 

The special application of biocompatible radioactive glasses for the treatment of liver cancer also deserves to be mentioned [60,61,62]. Insoluble Y_2_O_3_–Al_2_O_3_–SiO_2_ glass microspheres (diameter of 25 μm) with as much as 50 wt % yttrium oxide are injected into the patient’s blood flow, to lodge in the capillary bed of the diseased liver, which is a highly-vascularized organ. Before arterial infusion, the glass beads were bombarded by neutrons that create ^90^Y, a radioisotope that is a short-half-life (64 h) and short-range β-rays emitter. In this way, a localized dosage of up to 15,000 rad could be delivered towards malignant cells; this is a significant achievement considering that a maximum of 3000 rad can be tolerated by the patient under conventional external radiotherapy [63]. At present, radioactive glass microspheres (trade-named as TheraSphere^®^) are clinically used for the treatment of hepatocellular carcinoma and metastatic liver cancer in many specialized centers in North America and Europe after receiving FDA approval in 1999. This therapeutic approach leads to a significant improvement of survival times and quality of life for the patients.

In summary, the existing literature and current research trend suggest that the full potential of BGs in medicine is still yet to be fully exploited, and the relevant market is expected to further grow in the next few years. 

## 3. Grand Challenges for the Future—Where Are We Going?

**Figure 2:**
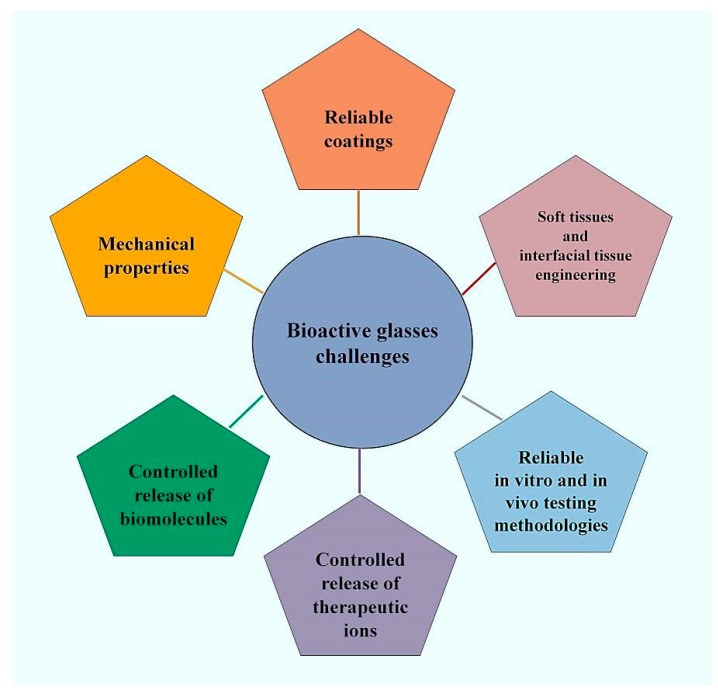
The most important challenges proposed for bioactive glasses (BGs) in medicine.

The impressive experimental research carried out over fifty years—since Hench’s early study in 1969 to date—has clearly demonstrated the great suitability and versatility of BGs in medicine, and has led to the development of many clinical products that improved the patient’s well-being and rehabilitation. This background of accumulated knowledge is expected to stimulate scientists to continue research and discover new applications for BGs in the effort to cope with the challenges of today’s society. This section provides an overview of what are, in the authors’ view, the six “hottest topics” related to BGs (see Figure 2) that will deserve to be tackled in the next future. 

### 3.1. Challenge No. 1: Reliable BG Coatings

This is perhaps the oldest challenge associated with the use of BGs in orthopedics and dentistry, where metallic implants are usually employed. It is known that, after being implanted, metals are prone to be encapsulated within fibrous tissue in the body; on the contrary, BG coatings have the potential to improve the implant stability by bonding it to the host bone and to protect the metallic substrate from corrosion, thus avoiding the release of toxic metal ions in vivo [64]. The major limitation of BG coatings is that they are, by nature, biodegradable, according to various dissolution rates that depend on the glass composition and environmental pH. As a result, a highly bioactive—i.e., reactive—BG coating may rapidly degrade over time, causing instability of the metallic implant lying underneath. This is probably the major reason why the use of BG coatings is limited compared to other bioceramics, such as non-resorbable thermal-sprayed hydroxyapatite [65].

An additional drawback of BGs used in the form of surface coatings is the mismatch between their thermal expansion coefficient (TEC) and that of the substrate on which they are applied. Ideally, the TEC of BG should approach that of the substrate to prevent the glass pulling away from the implant upon thermal processing (e.g., sintering) [66]. However, the TECs of 45S5 Bioglass^®^ (15 × 10^−6^ °C^−1^) and of most of silicate BGs are significantly higher than that of titanium alloys (about 9 × 10^−6^ °C^−1^), which are commonly used to produce prosthetic implants. 

Another crucial issue concerning BG coatings is the assessment of the long-term stability of the coatings in vivo. Surprisingly, there is still a paucity of contributions addressed to this topic in the literature. We found only one clinical study reporting the performance of BG-coated metallic femur stems (Biovetro^®^, (46–53)SiO_2_–(9–20)CaO–(7–24)Na_2_O–(0.1–2)MgO–(4–8)P_2_O_5_–(2–8)K_2_O–(0.1–2)Al_2_O_3_ mol %) [67]. A worse osteointegration was observed compared to plasma-sprayed hydroxyapatite coatings, the appearance of a fibrous interface with a macrophage foreign body reaction (probably due to the BG coating fragmentation), a significant delay in bone maturation, and an insufficient mineralization of the newly-formed bone. The authors of that study were perhaps discouraged by these partially negative results, and the research on Biovetro^®^-coated implants was apparently discontinued.

Therefore, a great challenge of the next few years will be the development of new BGs with more suitable TEC and dissolution rates for use as coating materials. Furthermore, new deposition techniques for BG coatings will have to be developed and/or optimized to improve the coating performance [68]. In the last years, new approaches (e.g., multilayer BG coatings to achieve a good compromise between adequate TEC, slow dissolution rate, and bioactivity [69]) and fabrication methods (e.g., electrophoretic deposition [70], radio-frequency sputtering [71]) have been experimented to produce well-adherent and durable coatings on a variety of materials and implants, including scaffolds, suture wires, surgical screws, and ocular implants. At present, it is impossible to state that one strategy is clearly preferable to another, and further research remains to be performed.

### 3.2. Challenge No. 2: Mechanical Properties—BG-Based Strong Scaffolds and Self-Healing Implants

Many biomaterials, including BGs, are often processed in the form of three-dimensional (3D) porous templates, i.e., implantable scaffolds, that can support and direct the regeneration of newly-formed healthy tissue. The fabrication of 45S5 Bioglass^®^-derived scaffolds was pioneered by Chen et al. in 2006 [39]. However, these early macroporous scaffolds produced by sponge replication were dramatically brittle (compressive strength within 0.3–0.4 MPa), and were then unsuitable for safe implantation in clinics. This limitation was due to an inherent drawback of 45S5 composition, i.e., its poor sinterability, which resulted in scaffolds with hollow struts (the inner channel in the strut was the “trace” of the sacrificial polymer template removed upon thermal treatment). The mechanical properties of foam-like BG scaffolds can be significantly improved either by applying a polymeric coating on the surface of the struts, or by properly tailoring the composition of the starting glass. In the first approach, the polymer layer acts as a “glue” that holds the BG particles together when the scaffold struts start to fail. The presence of a poly(3-hydroxybutyrate) (PHB) coating allowed the compressive strength of 45S5 Bioglass^®^ foams to increase up to 1.5 MPa, which is about three times the strength of the untreated scaffolds [72]. If a cellulose coating was used, the polymeric fibers were shown to perform a “bridging action” among the glass particles, and the scaffold failure was delayed [73]. The function of the polymer coating is essential to increase the toughness of the brittle BG porous substrate lying underneath. 

The second strategy of modifying the original 45S5 composition in order to improve the mechanical performance led to the development of many other BGs with a larger sinterability window (e.g., 13–93), which allowed strong scaffolds with well-densified struts to be obtained (compressive strength close to 20 MPa) [41]. 

A third approach involves the optimization of the thermal processes that are usually carried out to obtain BGs in a porous form. For example, it was reported that the compressive strength of 45S5 Bioglass^®^-derived foams can be increased to 2.5 MPa by optimizing the sintering temperature and time [40]. 

**Figure 3:**
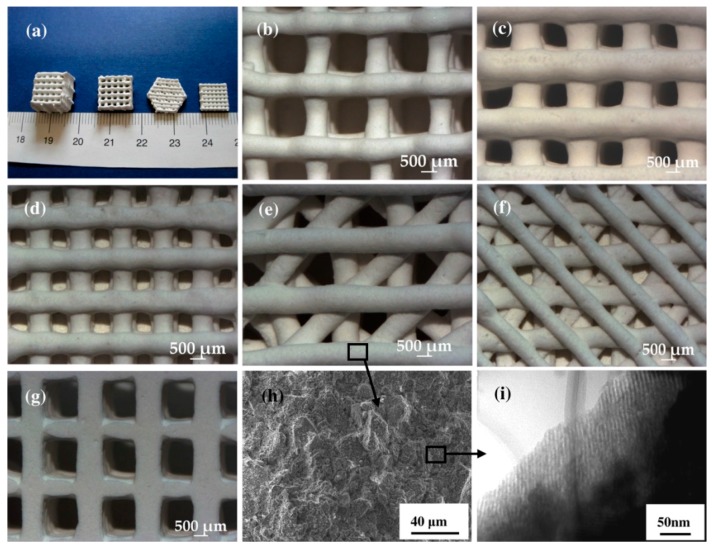
The representation of 3D printed mesoporous bioactive glass (MBG) scaffolds and their pore morphology and microstructure. (**a**) MBG scaffolds with different sizes, shapes, and morphologies. (**b**–**d**) The scaffolds with different pore sizes of (**b**) 1307 ± 40, to (**c**) 1001 ± 48, and (**d**) 624 ± 40 μm. (**d**–**f**) Different morphologies of MBG pore. (**g**) Pore morphology of the MBG from the bottom view scaffolds. (**h**) SEM micrograph of the microstructure of pore walls. (**i**) TEM image of the samples demonstrating the well-ordered mesopore channel structure of the pore walls. Reproduced with permission from Wu et al. [78].

In the last few years, additive manufacturing techniques (AMTs) have emerged as a valuable approach to process porous BGs, with porosity and mechanical comparable to the cancellous bone at a relatively reasonable cost [74]. Furthermore, AMTs are showing great promise for the fabrication of hierarchical scaffolds based on mesoporous BGs (MBGs), which exhibit an inherent nanoporous texture at the mesoscale (2–50 nm) [75]. Initially, hierarchical sol-gel MBG scaffolds were produced either by foaming approaches [76] or by dipping a macrocellular template (e.g., a polymeric sponge) into the sol [77]; however, very brittle structures were usually obtained, with poor compressive strength (less than 1 MPa). A tremendous improvement can be achieved by making use of AMTs. Wu et al. [78] applied 3D-printing to process SiO_2_–CaO–P_2_O_5_ MBG powder using poly(vinyl alcohol) as a binder, and obtained macro-mesoporous scaffolds with a compressive strength of 16 MPa, along with excellent mineralization ability and sustained drug release property (Figure 3). 3D-printed MBG scaffolds were also shown to retain good mechanical strength (7 MPa) after being soaked in simulated body fluids [79]. Limitations of AMTs that still remain to be overcome include the low feature resolution, which is still far from the size of living cells (typically few tens of micrometers), and the difficulty in manufacturing complex and highly delicate structures. On the other hand, a huge benefit showing great promise for the future is the ability to combine different biomaterials during the printing process, including soft phases (e.g., polymers), inorganic particles (e.g., BGs) and even cells, thus opening new horizons toward biofabrication of tissues and organs (see also the Section 3.3).

Natural tissues exhibit the unique ability of self-healing and repair, which synthetic implants do not have. A special class of recently-developed biomaterials, the so-called “hybrids”, are perhaps the very last frontier for obtaining implants with tissue-like properties [80]. Hybrid sol-gel materials are composed of interpenetrating networks of inorganic and organic phases, which are able to intimately interact at the nanoscale, thus allowing the whole material to behave as a single phase, unlike “conventional” nanocomposites [81]. This feature is responsible for highly controllable degradation rates, as well as adjustable mechanical properties, according to the specific application [82]. Furthermore, fine-scale dispersion of the components promotes enhanced interaction at the cellular level, resulting in rapid cell adhesion on the material surface [83]. The latest studies suggest that the most suitable inorganic phase for producing hybrid biomedical materials is represented by BGs, usually in the form of binary (SiO_2_–CaO) or ternary systems (SiO_2_–CaO–P_2_O_5_ or SiO_2_–CaO–Na_2_O), resulting in a valuable combination of bioactive behavior and highly congruent degradation rates [84,85,86]. An additional added value of hybrids was very recently announced by Julian Jones at the 28th Conference of the European Society for Biomaterials (ESB 2017) held in Athens on September 2017: early experiments conducted by his group at the Imperial College of London have shown that small cracks or flaws, observed in hybrid samples that underwent bending tests, tend to spontaneously self-repair within some hours from failure (the mechanical damage was no longer visible). The mechanism behind this very promising self-healing ability remains to be understood and will indeed motivate further research; the forthcoming publication of these exceptional results is expected with great interest by the scientific community. 

### 3.3. Challenge No. 3: Beyond Bone Repair—BGs in Contact with Soft Tissues and Interfacial Tissue Engineering 

The suitability of BGs for the repair of calcified tissues has been well established over fifty years of experimental research, which allowed biomaterials scientists to understand many of the biochemical and biological mechanisms behind BG–bone interaction. The emerging applications of BGs in contact with soft tissues have opened a new horizon that is still yet to be mostly explored.

There is highly encouraging experimental evidence that BGs can be potentially useful for a wide number of soft tissue engineering applications, such as wound dressing and the regeneration of cardiac, pulmonary, and gastrointestinal tissues [87,88]. At present, the “healing effect” of BGs on soft tissues is mainly attributed to improved angiogenesis, due to the release of ionic dissolution products from the glass. One of the greatest challenges that BG scientists will have to tackle in the next years is the in-depth understanding of the biomolecular mechanism behind the BG-induced angiogenesis, just as performed for bone applications in the last decades. This potential has been partially exploited in some early clinical applications (e.g., the “ReadiHeal” BG fibers, see Table 1 and Section 2): investigators have reported that a high calcium content in BG is a key factor in the wound healing of skin, and hypothesized that it is required for the migration of epidermal cells [53]. The release of Ca^2+^ ions is also suspected to play an important role in the late stages of healing, and the presence of calcium in the immediate vicinity of an open wound seems to help the body to regulate wound healing processes more effectively, particularly in open wounds. Doping with small amounts of selected ions, e.g., Cu^2+^ [89], can further potentiate the angiogenetic effect of BGs. Copper is known to regulate a number of factors involved in angiogenesis, such as vascular endothelial growth factor (VEGF), fibronectin, angiogenin, and fibroblast growth factor (FGF) 1 and 2, which play key roles in the initiation (vasodilation and vascular permeabilization), maturation (endothelial cell proliferation, migration and morphogenesis), and regulation of blood vessel formation (ECM remodeling) [90]. From a biomolecular viewpoint, two signaling pathways are involved in the Cu-induced angiogenesis: the first is associated to the Cu-activated hypoxia-inducible factor-1 (HIF-1), which acts in the initiation of angiogenesis process [91], and the second is the mitogen-activated protein kinase (MAPK) signaling pathway, which plays a key role in endothelial cell proliferation [92].

As recently reviewed by Kargozar et al. [93], many other ions have been described to locally stimulate angiogenesis—for example, Co^2+^ ions are associated with a hypoxia-mimicking mechanism and are a potent angiogenetic agent [94,95] but the interactions between these dopants and the cells, as well as their fate in the living organisms (accumulation, resorption via normal metabolism, excretion), are still yet to be fully elucidated (see also the Section 3.5). 

In this regard, a very crucial issue deserving careful future investigation is the risk of systemic toxicity induced by BGs to soft tissues and organs. Some studies performed in animal models (rabbits) with relative simple BG compositions (e.g., 45S5 Bioglass^®^) showed neither morphological damage to tissues nor accumulation of ions (especially Si) in key organs such as brain, heart, lungs, liver, kidney, and spleen [96,97,98]. Such studies are indeed expensive, time-consuming, and complex, as they require close collaboration among scientists from various disciplines (biomaterials scientists, bioengineers, biologists, histopathologists) but are essential to progress the research on BGs beyond current knowledge.

Another key issue related to the use of BGs in contact with soft tissue is inherent to their peculiar property of inducing the formation of a hydroxyapatite layer at the tissue/implant interface; hence, the risk of soft tissue calcification by BGs should be properly addressed and investigated in the future. 

The use of AMTs in processing BGs will be very valuable in the fields of soft tissue engineering and biofabrication, which combines biomaterials, biomolecules, and living cells as building blocks to print tissue and organs. The last frontier of AMTs applied to biofabrication is the simultaneous regeneration of multiple tissues by producing functionally-graded (FG) scaffolds for use in interfacial tissue engineering. Early experiments on the fabrication of heterogeneous organs (outer ear, kidney, and tooth) by using multi-head printing systems have been recently reported [99]; this strategy is currently limited to print soft matter (polymeric hydrogels and cells), but the incorporation of “rigid” BG inclusions is expected to be possible after some technological optimizations in the next future. Liverani et al. [100] prepared early prototypes of stratified scaffolds (porous BG/interfacial region/chitosan-alginate soft polymeric layer) for osteochondral tissue engineering by combining foam replication, freeze-drying, and electrospinning. 3D printing of BGs has the potential to meet this grand challenge and to further expand the applications of BGs from the restoration of bone and teeth to the repair of soft tissues and organs.

### 3.4. Challenge No. 4: Bioactive Glasses as Vehicles for the Controlled Release of Biomolecules

The potential of BGs for the delivery of various therapeutic biomolecules has been widely evaluated due to the possibility of incorporating both hydrophilic and hydrophobic groups into their structures [101]. In this regard, a broad spectrum of both natural and synthetic substances have been incorporated into BGs, in order to improve their biological activities (e.g., antibacterial effect) and, thereby, obtain an enhanced tissue repair and regeneration [102]. For example, Domingues et al. in a pioneering study, used BGs as a controlled release system for tetracycline hydrochloride and its inclusion complex made of tetracycline and b-cyclodextrin [103]. In this study, it was shown that the sol-gel method is useful for producing drug-containing BGs. However, the degradation of the loaded biomolecule by heat—e.g., during glass sintering—is considered as one of the main challenges. Furthermore, organic solvents used in the preparation of BGs are another factor that can cause denaturation of biomolecules (e.g., proteins). For instance, it has been shown that proteins are unstable in polar solvents like ethanol, which may be used in the sol-gel process [104].

Another challenge of BGs for drug delivery is associated with their degradable nature in biological environments. As mentioned in Section 3.1, the biodegradation of glass depends on its composition, as well as environmental pH, which directly affects the amount of drug released. With reference to this issue, Kim and colleagues could control the degradation and drug-release rate of vancomycin from phosphate glass/polycaprolactone composites through modifying the glass composition (0.45P_2_O_5_–xCaO–(0.55–x)Na_2_O, x = 0.2, 0.3, 0.4 or 0.5 mol %) [105]. Their results showed the drug release from the composites was strongly determined by the dissolution rates of the glasses, which is strongly related to the glass composition. 

Surface modification of BGs was also taken into account as another factor affecting drug release kinetics. In this regard, Farag and colleagues evaluated the influence of gamma-irradiation (25 and 50 kGy) on the release of vancomycin from nano-bioactive glass (NBGs, labelled as G25 and G50) [106]. They explained that diffusion was the main mechanism of drug delivery from the spherically-shaped NBG carrier. Their results revealed that the un-irradiated samples significantly adsorbed more drug amount (9.1 mg/0.2 g) in comparison to G25 and G50 samples (5.3 and 8.5 mg/0.2 g, respectively). The authors stated that this decrease in drug loading for the treated samples (G25 and G50) was caused by electrostatic repulsion forces established between negatively charged vancomycin molecules and increased non-bridging oxygens (NBOs) of the glass surface (i.e., increased the negative charge on the glass surface) as a result of gamma-irradiation. Hence, it should be noted that gamma-irradiation used in sterilization processes may greatly affect the drug-loading potential of the glasses.

As one of the latest members of the vast BG family, sol-gel derived MBGs have attracted much attention in the last decade. In fact, this special group of silicate materials was developed with the aim of becoming suitable carriers for therapeutic biomolecules. Compared to conventional BGs, MBGs possess high pore volume, high specific surface area, and uniform pore size, which make them suitable drug delivery systems. Moreover, the presence of a large number of Si-OH groups on the walls of mesoporous channels is identified as a facilitator for efficient drug loading, since these chemical groups can interact with target biomolecules via the hydrogen bond and van der Waals forces [107,108]. 

**Figure 4:**
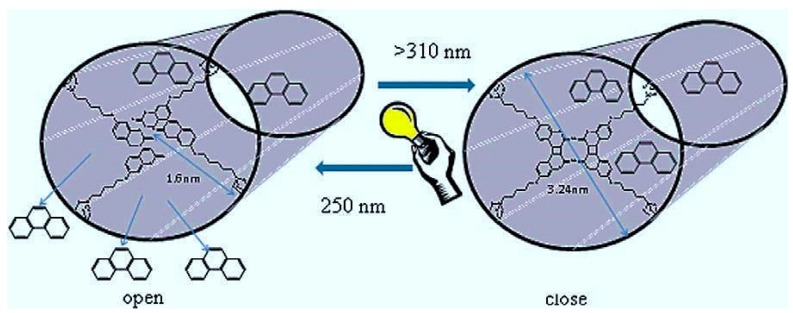
Controlled release of phenanthrene from the MBG modified using coumarin. UV light irradiation (>310 nm) induces photodimerization of the coumarin-modified MBG, which results in the pore closing with cyclobutane dimers, and trapping of the drug in the mesopores. On the other hand, the irradiation with shorter wavelength UV light (250 nm) leads to regenerate the coumarin monomer derivative through the photocleavage of cyclobutane dimers, and thereby, the trapped molecules are released from the mesopores. Reproduced with permission from Lin et al. [110].

In order to show the advantage of MBGs as compared to non-mesoporous glasses regarding drug delivery strategies, Zhu and coworkers prepared 3D MBG and conventional BG scaffolds with a composition of 80SiO_2_–15CaO–5P_2_O_5_ (mol %). Their results revealed that the efficiency of drug (gentamicin) loading for the MBG scaffold was two times higher than that of BG carriers. Furthermore, the release rate of gentamicin from the mesoporous scaffold was much lower than that from the BG scaffold. However, the quick release of biomolecules from MBG due to their open mesopore channels was considered a challenge in terms of sustained drug delivery strategies. Li and colleagues stated that this problem is the major barrier on the way of long-term use of MBGs as a drug carrier in orthopedic applications [109]. They proposed the development of novel composite microspheres of gentamicin-loaded MBG particles incorporated in a biodegradable poly(d,l-lactide-*co*-glycolide) (PLGA) matrix, and showed the usability of this system for the sustained release of the antibiotic. Another strategy was reported by Lin et al., who developed light-sensitive intelligent drug delivery systems of a photoresponsive coumarin derivative-modified MBG [110]. They took advantage of the photodimerization (by UV light > 310 nm) and photocleavage (by UV light around 250 nm) of coumarin-modified MBG, in order to close and open the pores, thus providing a mean for controlling the release of biomolecules (Figure 4). 

### 3.5. Challenge No. 5: BGs as Vehicles for the Controlled Release of Therapeutic Ions—Beyond the Pharmaceutical Approach

**Figure 5:**
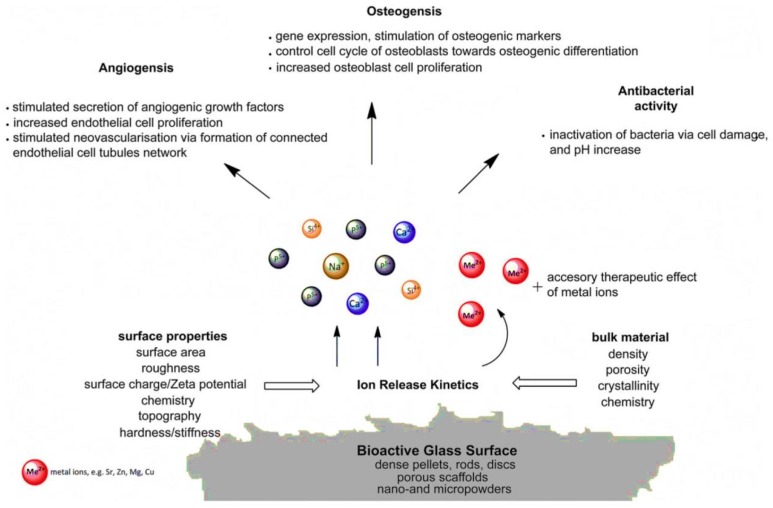
Schematic representation of biological responses to ionic dissolution products from bioactive glasses. (Reproduced with permission from Hoppe et al. [113]).

BGs are described to be able to exert their biological effects through releasing therapeutic ions into the environment (Figure 5). Up to now, a few trace elements (e.g., Sr, Cu, and Zn) have been added to BG structure for improving osteogenesis, angiogenesis, bactericidal activity, and anti-inflammation properties (see Table 2). However, it should be noted that these desired events are achievable if the ion release is controlled to an optimal concentration, allowing a suitable condition for human cells (ideally, non-toxicity). As previously well-documented, some of the trace metallic elements are potentially harmful to human cells and tissues at high concentrations, resulting in a challenge in this area. As an illustration, Kargozar et al. incorporated cobalt (Co^2+^) into the glass structure to stimulate angiogenesis [111]. Although this stimulation has been successful, the cytotoxicity of Co-containing glasses has increased as compared to the samples without Co^2+^ ions. In another study, Miguez-Pacheco and colleagues evaluated the biological effect of adding therapeutic niobium (Nb^5+^) ions to 45S5 BG [112]. Their results showed an increase in VEGF release as a valuable sign of angiogenesis in the case of the Nb-containing glasses. However, similar to cobalt, the cytotoxic effects of Nb-doped samples were higher than the control group. 

To date, a couple of factors have been recognized as determinant parameters regarding the degradation and subsequent release of ions from the glass structure to the environment. For example, Bruckner and colleagues investigated how the use of alkali ions of various ionic radii can affect the degradation rate of glass, and thereby, ion release from 45S5 Bioglass^®^ [114]. They showed that the inclusion of an alkali ion of smaller ionic radius (e.g., Li^+^ for Na^+^, or Na^+^ for K^+^) leads to a more compact glass network and, thereby, a decrease of ion release. By contrast, an alkali ion of larger ionic radius (e.g., K^+^ for Na^+^, or Na^+^ for Li^+^) results in an expansion in the silicate network of the glass, thus providing faster ion release.

The sintering temperature and pore network of scaffolds made of BGs are also identified as important players in the ion release. It has also been found that most of silicate-based BGs readily undergo devitrification during the processing of porous scaffolds as a result of sintering temperature. This crystallization at large amounts can result in reduced bioactivity of the glasses, as well as an uncontrolled release of ions [115]. For instance, Jones and colleagues have shown that an increase in the final sintering temperature from 600 to 800 °C leads to a decrease in dissolution rate of scaffolds and, thereby, reduced ions release [76]. In another study, the same research group explained that change in the pore network of scaffolds is another parameter influencing the release of ions from BGs [116]. Their results revealed that the rate of release of therapeutic ions (e.g., silicate and Ca^2+^ ions) is quicker for pore structures with a larger modal pore diameter. 

Based on the results of previously performed studies, it has been clarified that the addition of therapeutic ions into the BG structure and their subsequent release into the environment is usually not enough to gain the multifunctional properties needed for desired functions of cells and tissues. This issue is considered as a challenge on the way of therapeutic ions being incorporated in BG. On this matter, loading and delivery of various therapeutic agents, such as proteins, into BGs, has been become an alternative approach to achieve optimal results [117]. 

In general, there is a need for systematic approaches, the combination of in vitro and in vivo studies, and the use of bioreactors, in order to validate the effects of therapeutic ions under dynamic physiological conditions [118]. These proposed approaches and tools will certainly help to unveil the mechanisms of interaction between therapeutic ions released from BGs and human cells and tissues.

### 3.6. Challenge No. 6: BGs In Vitro and In Vivo—The Challenge of Having Reliable Testing Methodologies

**Figure 6:**
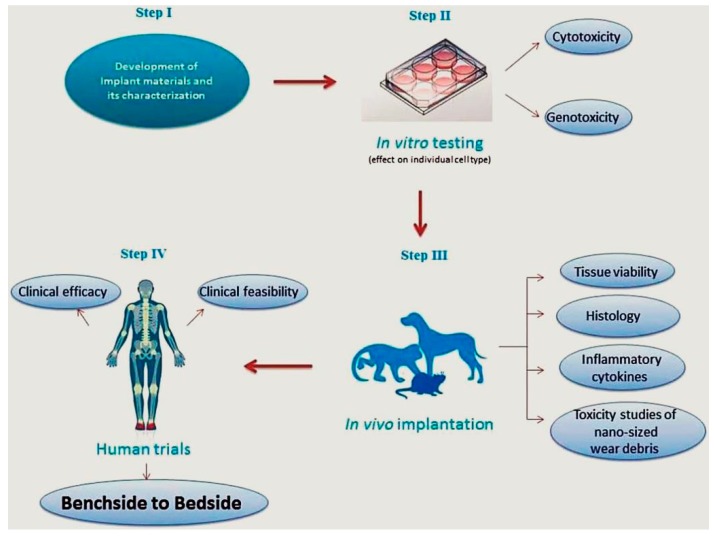
Representation of different steps involved in the translation of newly-developed biomaterials. Reproduced with permission from Thrivikraman et al. [153].

Following design, fabrication, and characterization of any material, it is necessary to evaluate its compatibility with the mammalian cells and tissues (Figure 6). This evaluation is conducted using some well-developed assays provided by the main international agencies, including FDA, European Medicines Agency (EMA), as well as regulatory bodies (i.e., ISO, ASTM, and USP) [152]. All these valid procedures, protocols, and standards are now available to assess all medical devices—including BGs—before implantation into the human body. All these developed procedures help researchers to save time and money prior to entering in vivo tests that are very time-consuming and expensive. 

It has been previously shown that any response of mammalian cells to the materials (e.g., BGs) nearly reflects the possible effects on the animal’s body. However, there are some critical challenges for the in vitro assays, as discussed in the following. Unfortunately, the use of appropriate cell lines for cytotoxicity evaluation is often not respected by researchers. They usually use fibroblasts instead of the cell lines similar to the tissues in implant sites: this approach is indeed useful for an early evaluation of the biocompatibility, but it should be corroborated by more advanced investigations. On the other hand, as stated in the literature [154], many of the cells used for confirming cytocompatibility are tumor-derived cell lines that do not necessarily represent the specific cells and tissues that may come in contact with the glasses. Use of primary cells from the patient could be the best option, but these cells are often very delicate, and ethical issues may also apply.

Another challenge is related to the incubation times applied by researchers, which are really short (commonly up to 7 days) to show biological (e.g., cytotoxic) effects of the glasses. The mammalian cells need enough time to attach, proliferate, and expand in vitro. Therefore, it seems necessary to assess the possible effects of released therapeutic ions for longer times, in order to confirm their compatibility as well as effectiveness. From the authors’ point of view, there are other issues that are usually ignored with respect to in vitro analyses, including the age of cells used, the reagents (e.g., cell culture media and supplements), and the conditions applied for their culture and expansion. 

Other limitations proposed for in vitro evaluation of BGs include lack of complexity, the absence of an immune or inflammatory response, and lack of the same cascade of events resulting from in vivo implantation (e.g., the interaction with components of blood). For example, the assessment of surface topography of scaffolds made of BGs is not possible by in vitro assays. In addition, in vitro tests are usually performed in a 2D system that cannot mimic the 3D environment of tissues and organs [155]. As a final point, the molecular mechanisms involved in the interactions of BGs and cells are often not checked by the researchers, which makes it difficult to draw a comprehensive conclusion about in vitro outcomes. 

As stated in a recently published article [155], the in vivo regenerative potential of BG-based scaffolds is determined by a number of factors, including their composition, fabrication approach, microstructure, and pore characteristics. Moreover, the pretreatment of BG scaffolds, as well as loading with cells and growth factors, could be effective regarding bone regeneration. 

The function of BGs and scaffolds made of them are evaluated through performing short and long-term in vivo experiments using small (rats [156,157,158] and rabbits [159,160]) and large (dog [161,162] and sheep [163,164]) laboratory animals. These models provide the assessment of the glasses under different conditions (e.g., load or non-load bearing) for extended time periods (from weeks to months). As mentioned in Section 3.2, the use of BGs for load bearing application is still limited, due to their mechanical property. Although this limitation has been partly solved after the emerging of 3D printing technology, BG-based scaffolds are still brittle, and not applicable for use at sites that are subjected to dynamic (often cyclical) loads [165].

Various bone defects, including calvarial, long bone, or maxillofacial defects, are created on the mentioned animals to show the potential of BGs with respect to osteocompatibility and osteogenesis. All the mentioned defects are categorized into non-critical and critical-sized defects. The primary criterion on the right selection of an animal model is its similarity with humans regarding physiological and pathological considerations [166]. However, the selection of the animal species, defect size, and implantation time could influence the in vivo behavior and performance of BGs. Moreover, comprehensive in vivo evaluations of the glasses used regarding accumulation, resorption via normal metabolisms, and excretion from the body are usually not carried out. 

As another challenge, the researchers are always faced with strict rules about the use of animals for in vivo evaluation of newly-developed BGs. Since laboratory animals may suffer from painful procedures during research, their use must be justified [167]. As suggested in the literature [161], a well-designed experiment helps to reduce the number of animals used in the research, so that scientists are allowed to collect data via the minimum number of animals needed. Though, it should be noted that a sufficient number of animals is needed to enable reliable statistical analysis and to generate significant results for preventing the repetition of additional experiments and the subsequent requirement to use more animals.

## 4. Conclusions

The abundant literature published until now on BGs witnesses the extraordinary versatility of these biomaterials, which primarily depends on the flexibility of their composition. The six grand challenges outlined above are mutually interlocked, as changing the glass composition to solve a specific problem (e.g., TEC adjustment, improvement of mechanical properties, incorporation of therapeutic ions) has an impact on other properties (e.g., bioactivity) that will change, too. This could be an advantage, as wise and careful design of glass composition allows tackling (and, hopefully, solving) multiple challenges at the same time: this will be the ultimate challenge for the next years. BG properties can also be tailored by acting on the fabrication process to produce, for example, macroporous scaffolds, mesoporous materials exhibiting drug release ability, or composite and multilayered constructs for interfacial tissue engineering. In summary, we forecast a bright future for the use of BGs in medicine, which will further expand the Glass Age. 

## Figures and Tables

**Table 1:** Chronology of the key applications of bioactive glasses in biomedicine.

Year (First Experimental Use)	Achievement/Application
**1969**	Invention of the 45S5 glass composition (45S5 Bioglass^®^)
**1977**	Treatment of ear diseases by using Ceravital^®^ glass-ceramics (replacement of middle ear small bones)
**1978**	Ocular implant (biocompatibility with corneal tissue)
**1985**	Approval by Food and Drug Administration (FDA) of the first 45S5 Bioglass^®^ implant (MEP^®^ implant for middle ear ossicular repair)
**1987**	Treatment of liver cancer (radioactive glasses)
**1988**	Clinical use of the 45S5 Bioglass^®^-based Endosseous Ridge Maintenance Implant (ERMI) in human patients
**1993**	FDA approval of PerioGlas (45S5 Bioglass^®^ particulate used for bone and dental repair)
**1998**	Peripheral nerve repair
**1999**	FDA approval of radioactive glasses (TheraSphere^®^) for cancer treatment
**2000**	Wound healing
**2002**	FDA approval of Medpor^®^-Plus^TM^ (polyethylene/45S5 Bioglass^®^ composite porous orbital implants).
**2003**	Antibacterial (Zn-containing) bone/dental cements
**2004**	Lung tissue engineering
**2004**	Use of mesoporous bioactive glass (MBG) as a drug delivery system
**2005**	Skeletal muscle and ligament repair
**2005**	Treatment of gastrointestinal ulcers
**2010**	Cardiac tissue engineering
**2011**	Commercialization of a cotton-candy borate bioactive glass for wound healing in veterinarian medicine. FDA approval is pending.
**2012**	Embolization of uterine fibroids
**2012**	Spinal cord repair
**2018**	Use of radioactive glasses (TheraSphere^®^) in patients with metastatic colorectal carcinoma of the liver

**Table 2:** The positive effects of the therapeutic ions released from BGs on the living cells and tissues.

Therapeutic ions		Biological Effects	Mechanism of Action	References
Monovalent	Silver (Ag)	Antibacterial activity	- Blocking the respiration and electron transfer as well as collapse the proton motive force in bacteria - Causing the leakage of massive proton through the bacteria cell membrane	[119]
	Lithium (Li)	Osteogenesis	- Activating Wnt/Catenin signaling pathway - Enhancing col1, Runx2, ALP, and bone sialoprotein	[120,121]
	Fluoride (F)	Osteogenesis	- Promoting Akt and GSK3β phosphorylation and activating the canonical Wnt/β-catenin signaling pathway - Stimulating the expression of bone differentiation markers of COL1A1, ALP, and osteonectin	[122]
Divalent	Calcium (Ca)	Osteogenesis	- Promoting SMAD signaling pathway	[123]
		Angiogenesis activity	- Increasing the expression of genes involved in angiogenesis including PDGF, EGF, IGF-I, bFGF, and VEGF - Inducing EC proliferation	[124]
	Strontium (Sr)	Osteogenesis	- Activating Wnt/Catenin signaling pathway - Up-regulation of genes expression of Runx-2, BMP-2, OCN, OPN, BSP, and Col1, ALP activity, and matrix mineralization - Enhancing attachment, proliferation, and differentiation of osteoblastic cells - Reduction of osteoclast activity	[125,126,127,128]
	Manganese (Mn)	Osteogenesis	- Upregulation of Runx-2 and OPN	[129]
		Antibacterial activity	- Generating ROS, and thereby inhibiting the bacteria germination of bacteria	[130]
	Magnesium (Mg)	Osteogenesis	- Activation of Notch1 signaling pathway	[131]
		Angiogenesis activity	- Overexpression of COL10A1 gene	[132]
	Zinc (Zn)	Osteogenesis	- Stimulating PKC/MAPK signaling pathways	[133]
		Antibacterial activity	- Enhancing the production of ROS, and thereby cause DNA, RNA, and protein damage - Destabilization of bacterial membranes	[134]
		Anti-inflammation activity	- Decreasing the expression of TNF-α, IL-1β, and VCAM by inhibition of NF-κB activation via A20 and PPAR-α pathways	[135]
	Copper (Cu)	Osteogenesis	- Activation of bone metabolism via the action as a cofactor for lysyl oxidase - Inhibiting bone resorption through the action as a cofactor for superoxide dismutase	[136]
		Angiogenesis	- Stabilization of nuclear HIF-1 a and simulating hypoxia, thereby activating proangiogenic factors VEGF, bFGF, TNF-α, and IL-1	[91]
		Antibacterial activity	- Attaching to the bacteria plasma membrane and making lethal changes in the cell membrane, such as disruption of membrane integrity inevitably	[137]
	Cobalt (Co)	Angiogenesis	- Inducing HIF and thereby the upregulation of angiogenic factors VEGF and bFGF	[95,138]
Trivalent	Cerium (Ce)	Osteogenesis	- Activation of TGF-β/BMP and Smad1/5/8 signaling pathway and thereby upregulation of genes of Runx 2, Col I, BMP2, ALP, and OCN	[139,140]
		Antibacterial activity	- Increasing the levels of ROS in the cerium-incubated bacteria cells, resulting in DNA, RNA, and protein damage	[141]
	Gallium (Ga)	Osteogenesis	- Inhibiting the differentiation and the resorbing activity of osteoclasts	[142]
		Antibacterial activity	- Inhibiting essential biological reactions of bacteria	[143]
	Boron (B)	Osteogenesis	- Activating MAPK signal pathway	[144]
		Angiogenesis	- Upregulation of VEGF and TGF-β1 genes	[145]
	Iron (Fe)	Osteogenesis	- Activating MAPK signal pathway - Upregulating Runx2, ALP, and BMP2 genes	[146]
	Europium (Eu)	Angiogenesis	- Overexpression of angiogenic genes of CD31, MMP9, VEGFR1/2, and PDGFRa/b	[147]
Tetravalent	Silicon (Si)	Osteogenesis	- Activation of BMP2 signaling pathway	[148]
		Angiogenesis activity	- Inducing endothelial cell (EC) homing, cell polarization, migration	[149]
Pentavalent	Phosphate (P)	Osteogenesis	- Stimulating the expression of matrix gla protein (MGP)	[150]
		Angiogenesis activity	- Stimulation of pro-angiogenic FOXC2, osteopontin, and VEGFa	[151]
	Niobium (Nb)	Angiogenesis	- Enhancing the secretion of VEGF	[112]
